# Effect of neuraminidase inhibitor (oseltamivir) treatment on outcome of hospitalised influenza patients, surveillance data from 11 EU countries, 2010 to 2020

**DOI:** 10.2807/1560-7917.ES.2023.28.4.2200340

**Published:** 2023-01-26

**Authors:** Cornelia Adlhoch, Concepción Delgado-Sanz, AnnaSara Carnahan, Amparo Larrauri, Odette Popovici, Nathalie Bossuyt, Isabelle Thomas, Jan Kynčl, Pavel Slezak, Mia Brytting, Raquel Guiomar, Monika Redlberger-Fritz, Jackie Maistre Melillo, Tanya Melillo, Arianne B. van Gageldonk-Lafeber, Sierk D. Marbus, Joan O’Donnell, Lisa Domegan, Joana Gomes Dias, Sonja J. Olsen

**Affiliations:** 1 European Centre for Disease Prevention and Control (ECDC), Stockholm, Sweden; 2 National Centre of Epidemiology, CIBERESP, Carlos III Health Institute, Madrid, Spain; 3 Public Health Agency of Sweden, Stockholm, Sweden; 4 National Institute of Public Health Romania-National Centre for Communicable Diseases Surveillance and Control, Bucharest, Romania; 5 Sciensano, Brussels, Belgium; 6 Department of Infectious Diseases Epidemiology, National Institute of Public Health, Prague, Czechia; 7 National Influenza Reference Laboratory, National Institute of Health Dr. Ricardo Jorge, Lisbon, Portugal; 8 Center for Virology, Medical University Vienna, Vienna, Austria; 9 Infectious Disease prevention and Control unit, Malta; 10 National Institute for Public Health and the Environment (RIVM), Bilthoven, the Netherlands; 11 Health Service Executive-Health Protection Surveillance Centre, Dublin, Ireland; 12 WHO Regional Office for Europe, Copenhagen, Denmark

**Keywords:** EU, fatal outcome, Influenza, hospital, antiviral treatment, risk factors, influenza virus, surveillance, clinic, epidemiology

## Abstract

**Background:**

Timely treatment with neuraminidase inhibitors (NAI) can reduce severe outcomes in influenza patients.

**Aim:**

We assessed the impact of antiviral treatment on in-hospital deaths of laboratory-confirmed influenza patients in 11 European Union countries from 2010/11 to 2019/20.

**Methods:**

Case-based surveillance data from hospitalised patients with known age, sex, outcome, ward, vaccination status, timing of antiviral treatment, and hospitalisation were obtained. A mixed effect logistic regression model using country as random intercept was applied to estimate the adjusted odds ratio (aOR) for in-hospital death in patients treated with NAIs vs not treated.

**Results:**

Of 19,937 patients, 31% received NAIs within 48 hours of hospital admission. Older age (60–79 years aOR 3.0, 95% CI: 2.4–3.8; 80 years 8.3 (6.6–10.5)) and intensive care unit admission (3.8, 95% CI: 3.4–4.2) increased risk of dying, while early hospital admission after symptom onset decreased risk (aOR 0.91, 95% CI: 0.90–0.93). NAI treatment initiation within 48 hours and up to 7 days reduced risk of dying (0–48 hours aOR 0.51, 95% CI: 0.45–0.59; 3–4 days 0.59 (0.51–0.67); 5–7 days 0.64 (0.56–0.74)), in particular in patients 40 years and older (e.g. treatment within 48 hours: 40–59 years aOR 0.43, 95% CI: 0.28–0.66; 60–79 years 0.50 (0.39–0.63); ≥80 years 0.51 (0.42–0.63)).

**Conclusion:**

NAI treatment given within 48 hours and possibly up to 7 days after symptom onset reduced risk of in-hospital death. NAI treatment should be considered in older patients to prevent severe outcomes.

**Key public health message**

**What did you want to address in this study?**

We aimed to provide more evidence of the effect and timing of antiviral treatment on severity and outcome in hospitalised patients with influenza virus by using routine surveillance data over a 10-season period. This knowledge should provide guidance and improve the clinical treatment of influenza patients with or without underlying conditions and clinical complications.

**What have we learnt from this study?**

Influenza can be severe, and early treatment opportunities to prevent severe outcomes were possibly missed. Of the hospitalised patients, 28% required intensive care and 14% died. While the majority (78%) of patients were given neuraminidase inhibitors, only 58% received antiviral treatment within the first 4 days and 30% within the first 48 hours as recommended.

**What are the implications of your findings for public health?**

Our results support the role of antiviral treatment in hospitalised patients with laboratory-confirmed influenza. Antiviral treatment was found to reduce the risk of in-hospital death when given early after influenza virus confirmation, but even when the 48-hour window after symptom onset has passed.

## Introduction

Influenza viruses pose a continuous threat to public health due to their ability to cause epidemics and pandemics [1,2]. Therefore, global surveillance is in place to constantly monitor and assess the epidemiological situation. The World Health Organization (WHO) emphasises the need to monitor the clinical severity of infected persons and collect data on underlying comorbidities to identify risk groups or conditions contributing to severity and mortality [3]. In addition to underlying conditions, virus type and age contribute to disease progression. Severity and impact of annual influenza epidemics are key factors used to assess the seasonal situation and guide interventions. Influenza-associated hospitalisations and fatalities are important indicators of the epidemiological situation and these data are routinely used by clinicians and public health experts to guide preventive measures.

Besides the availability of seasonal influenza vaccines that are updated annually, antiviral treatment with neuraminidase inhibitors (NAI) such as oseltamivir and zanamivir, M2 blockers (amantadine or rimantadine) or a cap-dependent endonuclease inhibitor (baloxavir marboxil) are available in Europe [4-9]. However, all seasonal influenza viruses are resistant to M2 blockers so this treatment option is not available. Baloxavir marboxil received market authorisation in Europe in January 2021 and has shown to shorten the duration of symptoms as well as prevent infections when given prophylactically [5]. Clinical studies have shown that oseltamivir and zanamivir shorten the duration of symptoms and the length of stay in hospitalised patients, prevent death in hospitalised patients with severe disease and possibly reduce influenza virus transmission [10-15]. Despite the documented benefits of antiviral treatment, it is infrequently prescribed for out-patient influenza cases. However, treatment is common in hospital settings [16-18].

European countries report case-based surveillance data on hospitalised influenza patients on a weekly basis to The European Surveillance System (TESSy) hosted at the European Centre for Disease Prevention and Control (ECDC). Data are analysed and basic indicators are published weekly in an aggregated form during the influenza season at www.FluNewsEurope.org [19]. In a more detailed study, these data have been used to assess the impact of underlying conditions on severe outcome but not to address the impact of antiviral treatment [20]. Improved reporting and data completeness has enabled the inclusion of these data into this analysis.

This study aims to assess the impact of antiviral treatment on in-hospital deaths of laboratory-confirmed influenza patients. These findings are important for public health communication and in guiding targeted prevention strategies of hospitalised severely ill patients.

## Methods

### Data and variables

The ECDC collects weekly case-based data on hospitalised laboratory-confirmed influenza cases from European Union (EU) and European Economic Area (EEA) countries through TESSy. Hospital surveillance and data collection are organised nationally by each country’s respective public health authorities. The public health authorities then report these data to TESSy following the outlined variables in the reporting protocol. However, the reporting protocol does not provide detailed clinical criteria for reporting underlying conditions, and only broadly states how to report underlying conditions such as chronic kidney disease, leaving it to individual countries to control the reporting. These data were downloaded from TESSy in week 34 2021 (snapshot 23 August 2021).

The weekly reported data contains the following variables for analysis: age, sex, onset of symptoms (if missing, date of notification), date of hospitalisation, virus (sub)type, antiviral treatment (none, oseltamivir, zanamivir, both, other), date of antiviral treatment, influenza vaccination status (for respective season), hospital ward (non-intensive care or intensive care units (ICU)), underlying conditions (asthma, cancer, diabetes, chronic heart, kidney, liver or lung disease, HIV/immunosuppression, neurocognitive or neuromuscular disease, obesity (body mass index (BMI) of 30 to < 40 kg/m^2^) or morbid obesity (BMI ≥ 40 kg/m^2^), pregnancy or other conditions (other diseases, smoking, genetic conditions e.g. trisomy), clinical complications (acute respiratory distress syndrome (ARDS), bronchitis, encephalitis, myocarditis, pneumonia, sepsis, other), date of discharge or death, and outcome (death or recovery). We combined underlying conditions (obesity and morbid obesity, neuromuscular and neurocognitive disorders, asthma and lung) when the numbers were small. Data inclusion and management of the free text field used is detailed in the Supplementary material.

Time between hospital admission and either discharge or death was used to calculate the duration of hospitalisation. The number of days between onset of symptoms and hospitalisation was calculated as well as the number of days between onset of antiviral treatment and hospitalisation. Initiation of antiviral treatment was calculated in relation to symptom onset. We classified the time points of antiviral treatment after symptom onset and used periods of within 48 hours, after 3–4 days, 5–7 days or > 7 days.

### Inclusion and exclusion criteria

Data reported during each influenza season between weeks 40 and 20 were included starting from week 40 2010 and ending week 20 2020. No hospitalised patient was reported during the 2020/21 season due to the impact of the coronavirus disease (COVID-19) pandemic. The analysis included only cases with complete reporting on sex, age, antiviral treatment, hospital ward, influenza vaccination status and outcome. The analysis included laboratory confirmed influenza cases reported as influenza A unsubtyped, A(H1N1)pdm09, A(H3N2) or type B without lineage, B/Victoria or B/Yamagata virus.

We excluded cases with hospitalisation or treatment initiation date before symptom onset (< 0 days), or > 21 days after symptom onset. Also excluded were cases where antiviral treatment, onset of treatment, hospital ward, outcome or vaccination status was reported as ’unknown’ or ’other’.

### Statistical analysis

The primary outcome measure was reported in-hospital death, and patients treated with antivirals were compared to untreated patients.

We performed a descriptive analysis of the pooled data and statistics included absolute numbers, mean, median and relative frequencies of each variable. A two-sided chi-squared test was used for categorical variables and continuous variables were compared using two-sided Student’s t-test (Wilcoxon rank-sum test when normality was not met).

We compared cases with antiviral treatment (oseltamivir, zanamivir or both) to untreated and stratified the time point of the antiviral treatment (within 48 hours or after 3–4 days, 5–7 days or ≥ 7 days after symptom onset). We performed a stratified analysis by age group and hospital ward (ICU vs non-ICU). Additional post-hoc analyses to the original study protocol were performed.

To identify significant determinants for fatal outcome, a multivariable mixed-effect logistic regression model was fitted using country as random intercept and sex, age group, influenza subtype, hospital ward and time of hospital admission as covariables. An analysis was performed to assess determinants for fatal outcome (death) known to impact mortality (lung, heart, kidney, liver disease, HIV/immunosuppression, cancer, diabetes, obesity, neuromuscular/cognitive disorders, ARDS, pneumonia and sepsis) and included a comparison of cases that received antiviral treatment within 48 hours after symptom onset to later treatment. The majority of vaccinated cases (89%, 5,732/6,417) were 60 years or older which is in accordance with the recommendation that people 65 years or older and risk groups with underlying conditions such as respiratory, cardiovascular, hepatic, renal, or neurological diseases, HIV/immunosuppression, metabolic or haematological disorders as well as morbid obesity be vaccinated [21]. Therefore, vaccination status was only included in the age-stratified and determinants analysis. To assess the effect of pregnancy, the dataset was restricted to women ≥ 14 to 49 years of age in a sub-analysis. Adjusted (for country as random intercept) odds ratios (aOR) and 95% confidence intervals (CI) were calculated. P values < 0.05 were considered significant. Stata 17 (StataCorp, College Station, United States) was used for all statistical analysis.

### Sensitivity analysis

We used three different approaches. Firstly, we propensity matched cases by age, sex, ICU or non-ICU admission and reporting country to balance between the treated and untreated group, and analysed with a conditional logistic regression. Secondly, we excluded the Spanish data to account for a country dominant effect and thirdly, we recoded excluded cases with missing data on antiviral treatment to include them as untreated. A mixed-effect logistic regression model including the country as random intercept was used for analysis (Supplementary Figure F5 showing the flow chart of the recoded analysis and Tables T9-T12 the results of the matched (with or without Spanish data) and recoded logistic regression analysis).

## Results

**Figure 1:**
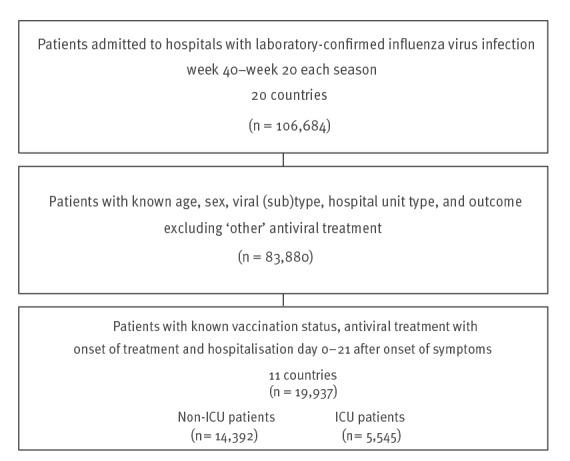
Flowchart of the included and excluded data on laboratory-confirmed hospitalised influenza patients, 11 European Union countries, influenza seasons 2010/11–2019/20

Of 106,684 cases reported from hospital settings, 19,937 fulfilled the inclusion criteria (Figure 1). Cases were reported from 11 countries (Austria, Belgium, Czechia, Ireland, Malta, the Netherlands, Portugal, Romania, Slovakia, Spain, Sweden) between 2010/11 and 2019/20. The level of reporting varied by each country each year, with Spain reporting 83% of cases. The Supplementary Table T1 shows the reported recovered and fatal cases by countries and year. Slightly more men than women were included (53.5% vs 46.5%) and the mean age of all cases was 60 years (median 67, Table 1). Men were slightly younger than women (median 66 vs 68 years, p < 0.001).

ICU: intensive care unit.

Participating European Union countries: Austria, Belgium, Czechia, Ireland, Malta, the Netherlands, Portugal, Romania, Slovakia, Spain, Sweden.

**Table 1:** Risk and protective factors for fatal outcome among hospitalised patients with influenza virus infections, descriptive and multivariable analysis, 11 European Union countries, influenza seasons 2010/11–2019/20

Variables	Outcome				Total		Multivariable analysis		
	Recovered		Death				aOR	95% CI	
	n	%	n	%	n	%			
Sex									
Female	7,982	46.7%	1,296	45.6%	9,278	46.5%	Ref.		
Male	9,115	53.3%	1,544	54.4%	10,659	53.5%	1.06	0.98–1.16	
Age group (years)									
0–19	2,323	13.6%	54	1.9%	2,377	11.9%	0.29^a^	0.20–0.41	
20–39	1,253	7.3%	98	3.5%	1,351	6.8%	Ref.		
40–59	3,573	20.9%	416	14.6%	3,989	20.0%	1.55^a^	1.22–1.97	
60–79	5,903	34.5%	1,070	37.7%	6,973	35.0%	2.99^a^	2.38–3.76	
≥ 80	4,045	23.7%	1,202	42.3%	5,247	26.3%	8.32^a^	6.57–10.54	
Influenza virus									
Type B	3,317	19.4%	533	18.8%	3,850	19.3%	Ref.		
A(H1N1)pdm09	4,511	26.4%	830	29.2%	5,341	26.8%	1.31^a^	1.15–1.50	
A(H3N2)	3,154	18.4%	601	21.2%	3,755	18.8%	0.99	0.87–1.14	
A unsubtyped	6,115	35.8%	876	30.8%	6,991	35.1%	0.88^b^	0.78–1.00	
Vaccination^c^									
Not vaccinated	11,790	69.0%	1,730	60.9%	13,520	67.8%	NA	NA	
Vaccinated	5,307	31.0%	1,110	39.1%	6,417	32.2%	NA	NA	
Hospital unit									
Non-ICU	12,832	75.1%	1,560	54.9%	14,392	72.2%	Ref.		
ICU	4,265	24.9%	1,280	45.1%	5,545	27.8%	3.76	3.40–4.17	
AV treatment									
No treatment	3,687	21.6%	626	22.0%	4,313	21.6%	NA	NA	
Oseltamivir, zanamivir or both	13,410	78.4%	2,214	78.0%	15,624	78.4%	NA	NA	
Timing of AV treatment									
No treatment	3,687	21.8%	626	22.3%	4,313	21.9%	Ref.		
Within 2 days	4,067	24.0%	700	25.0%	4,767	24.2%	0.51^a^	0.45–0.59	
3–4 days	3,618	21.4%	584	20.8%	4,202	21.3%	0.59^a^	0.51–0.67	
5–7 days	3,520	20.8%	523	18.7%	4,043	20.5%	0.64^a^	0.56–0.74	
> 7 days	2,038	12.0%	368	13.1%	2,406	12.2%	1.00	0.85–1.19	
Timing of hospitalisation	Mean (SD)	Median (IQR)	Mean (SD)	Median (IQR)	Mean (SD)	Median (IQR)		aOR	95% CI
Age in years	58.0 (26.8)	65 (34)	72.2 (17.9)	76 (23)	60.0 (26.2)	67 (32)		NA	NA
Time between onset of symptoms and hospitalisation in days	3.8 (3.1)	3 (3)	3.3 (3.0)	3 (4)	3.7 (3.1)	3 (4)		0.91^a^	0.90–0.93
Duration of hospitalisation in days	9.6 (11.9)	6 (8)	14.0 (20.7)	8 (12)	11.0 (15.2)	7 (10)		NA	NA
Time between onset of symptoms and antiviral treatment in days	4.6 (3.5)	4 (4)	4.6 (3.7)	4 (4)	4.6 (3.5)	4 (4)		NA	NA

aOR: adjusted odds ratios using a mixed effect model adjusted for country as random intercept; AV: antiviral; CI: confidence interval; HIV/immunos.: HIV infection or immunosuppression; ICU: Intensive care unit; IQR: interquartile range; NA: not analysed; Ref.: reference group; SD: standard deviation.

^a^ p < 0.001.

^b^ p < 0.05.

^c^ Only included in age group stratified analysis because it is only recommended for risk groups and > 65-year-olds [21].

Participating European Union countries: Austria, Belgium, Czechia, Ireland, Malta, the Netherlands, Portugal, Romania, Slovakia, Spain, Sweden.

Of the hospitalised patients, 27.8% (n = 5,545) were admitted to ICU, with some countries reporting ICU cases only. A higher proportion of men than women (57.9% vs 42.1%) were admitted to ICU compared to non-ICU (51.8 vs 48.2%) (p < 0.0001). A higher proportion of patients died in the ICU compared with non-ICU (23.1% vs 10.8%, p < 0.0001).

The median time between symptom onset and hospitalisation was 3.6 days and was slightly shorter in fatal cases than in survivors (3.3 vs 3.8 days). Duration of hospitalisation was available for 1,930 (9.7%) of patients, with a median stay of 7 days (mean 11 days) (Table 1). Duration was similar for men and women (p = 0.15) and for patients with influenza A and B virus infection (p = 0.27). However, duration of hospital stay was significantly longer for patients who died (median 8 days) compared with those who recovered (median 6 days, p < 0.0001) as well as for patients in the ICU (median 10 days) compared with non-ICU patients (median: 6 days; p < 0.0001, Supplementary Table T3 providing a descriptive overview of patient characteristics stratified by ICU and non-ICU ward).

In-hospital death was reported for 14.2% (2,840) of patients, and 54.9% of these deaths were in non-ICU. Patients who died were significantly older than those who survived (median age 76 vs 65 years, p < 0.0001). Of those who died, 54.4% (1,544) were male, which was a similar proportion to the group that recovered (53.3%, p = 0.3) (Table 1).

Vaccinated patients were older than unvaccinated (mean age: 75.4 vs 52.7 years, p < 0.0001). Of the 5,732 vaccinated cases, 89% were 60 years or older and 48% were 80 years or older.

### Influenza virus type, subtype or lineage

Influenza A virus infection was detected in 80.7% of patients (Table 1). Among the 9,096 patients with known influenza A virus subtype, slightly more A(H1N1)pdm09 (58.7%) than A(H3N2) (42.3%) virus infections were reported. The vast majority of influenza type B viruses (91.2%, 3,513/3,850) were reported without lineage; 333 infections were reported as B/Yamagata and four as B/Victoria so all influenza type B virus cases were pooled for further analysis. The lowest proportion of influenza type B compared with influenza type A viruses was observed in 40–59-year-olds (13.5%) and 20–39-year-olds (15.6%), while the highest was seen in the youngest (0–19 years, 23.0%) and oldest age group (≥ 80 years, 22.5%). The highest proportion of cases due to influenza A(H3N2) virus infection were in patients 80 years or older (26.3%) while the highest proportion of cases with A(H1N1)pdm09 virus infection were patients 20–39 years old (44.7%) and 40–59 years old (41.5%).

The virus distribution among patients in the ICU and non-ICU was different, with a lower proportion of influenza type B (16.6% vs 20.4%) and A(H3N2) viruses (16.6% vs 19.7%) and higher proportion of A(H1N1)pdm09 (35.3% vs 23.5%, p < 0.0001) in patients admitted to the ICU than non-ICU patients, respectively (Supplementary Table T3 providing a descriptive overview of patient characteristics stratified by ICU and non-ICU ward). Patients who were admitted to the ICU were younger than those in non-ICU wards (mean age 54.3 vs 62.2 years, p < 0.0001).

### Antiviral treatment

**Figure 2:**
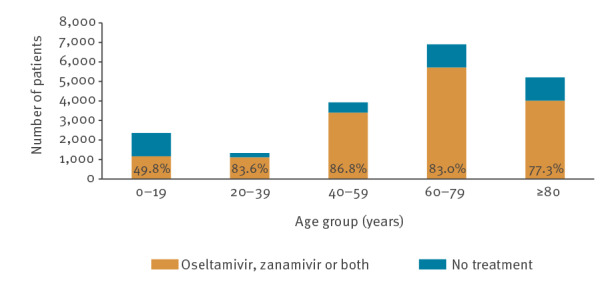
Number and proportion (%) of hospitalised influenza patients receiving antiviral treatment by age group, 11 European Union countries, influenza seasons 2010/11–2019/20

Overall, 78.1% of patients admitted to hospital received antiviral treatment. Of these, 98.3% received oseltamivir, a few cases were treated with zanamivir (0.3%) or a combination of both (0.1%), and 206 (1.3%) had unspecified antiviral treatment. The highest proportion receiving antiviral treatment (86.8%) was in patients aged 40–59 years (Figure 2). About one third (31%) of patients received NAIs within 48 hours, 58% received NAIs within the first 4 days and 84% within 7 days of symptom onset (Supplementary Table T2 showing a descriptive overview of patient characteristics by NAI treatment or no treatment). A small proportion of patients received NAI treatment before being admitted to hospital (4.9%, 769/15,628). The treatment started a median of 4 days (mean 4.6 days) after symptom onset (Table 1). A higher proportion of ICU patients were NAI-treated compared with non-ICU patients (85.6% vs 75.3%, p < 0.00001).

Participating European Union countries: Austria, Belgium, Czechia, Ireland, Malta, the Netherlands, Portugal, Romania, Slovakia, Spain, Sweden.

### Multivariable analysis

Increasing age had a statically significant impact on patient outcome with children and young adults (0–19 years) less likely to die compared with the reference group of 20–39-year-olds. Additionally, patients 80 years or older had 8.32 times higher odds of dying (Table 1). The earlier people were admitted to hospital after symptom onset, the less likely they were to die (aOR 0.91, 95% CI: 0.90–0.93), while admission to ICU and influenza A(H1N1)pdm09 virus infections were associated with an increased risk of dying (aOR 3.76, 95% CI: 3.4–4.2 and aOR 1.3, 95% CI: 1.2–1.5, respectively). Neuraminidase inhibitor treatment initiated up to 7 days after symptom onset was associated with a lower risk of death compared with untreated cases, and later treatment commencement showed no effect. The risk of in-hospital death was lower in all NAI-treated patients compared with untreated (aOR 0.62, 95%CI: 0.56–0.80, model run without treatment timing).

### Stratified analysis by age group and hospital ward

An age group stratified analysis confirmed that ICU admission was strongly associated with risk of death across all age groups (Table 2). Infection with influenza A(H1N1)pdm09 virus was identified as a risk factor in 0–19 and 40–59-year-olds. Earlier hospitalisation after symptom onset was protective in all age groups apart from the youngest. While antiviral treatment had no impact on outcome in 0–19 and 20–39-year-olds, treatment lowered the risk of death when initiated up to 7 days after onset of symptoms in all other age groups, and even after 7 days for patients 80 years or older. Vaccination, included in the oldest age groups, did not show any effect on outcome.

**Table 2:** Risk of death in hospitalised influenza cases by age group, sex, influenza subtype, timing of antiviral treatment, intensive care unit admission status, timing of hospitalisation and vaccination status, 11 European Union countries, influenza seasons 2010/11–2019/20

Variable	0–19 years		20–39 years		40–59 years		60–79 years		≥ 80 years	
	aOR	95% CI	aOR	95% CI	aOR	95% CI	aOR	95% CI	aOR	95% CI
Female sex	Ref.	Ref.	Ref.	Ref.	Ref.	Ref.	Ref.	Ref.	Ref.	Ref.
Male sex	0.81	0.45–1.46	1.16	0.73–1.83	1.06	0.84–1.33	1.16^a^	1.00–1.33	1.02	0.89–1.16
Influenza virus										
Type B	Ref.		Ref.		Ref.		Ref.		Ref.	
A(H1N1)pdm09	2.65^a^	1.24–5.66	1.23	0.59–2.57	2.09^b^	1.40–3.13	1.18	0.95–1.45	1.13	0.90–1.42
A(H3N2)	0.41	0.11–1.54	1.07	0.41–2.81	1.48	0.89–2.45	0.91	0.73–1.14	1.01	0.84–1.22
A unsubtyped	1.05	0.38–2.89	0.89	0.38–2.09	1.38	0.89–2.11	0.81^a^	0.66–0.99	0.85	0.72–1.02
Timing of AV treatment										
No treatment	Ref.		Ref.		Ref.		Ref.		Ref.	
Within 2 days	1.27	0.55–2.93	1.38	0.58–3.29	0.43^b^	0.28–0.66	0.50^b^	0.39–0.63	0.51^b^	0.42–0.63
3–4 days	1.13	0.46–2.80	1.15	0.48–2.75	0.62^a^	0.42–0.92	0.52^b^	0.41–0.65	0.60^b^	0.49–0.73
5–7 days	1.08	0.36–3.18	1.55	0.64–3.78	0.64^a^	0.43–0.94	0.59^b^	0.47–0.75	0.65^b^	0.52–0.81
> 7 days	2.90	0.99–8.55	3.02^a^	1.05–8.68	1.16	0.75–1.82	1.07	0.81–1.41	0.72^a^	0.54–0.96
Non-ICU admission	Ref.	Ref.	Ref.	Ref.	Ref.	Ref.	Ref.	Ref.	Ref.	Ref.
ICU admission	13.23^b^	6.05–28.92	14.75^b^	7.79–27.92	6.28^b^	4.84–8.14	4.13^b^	3.56–4.78	1.49^c^	1.17–1.89
Time between symptom onset and hospitalisation	0.90	0.79–1.04	0.84^c^	0.74–0.95	0.91^b^	0.86–0.95	0.91^b^	0.88–0.94	0.93^b^	0.90–0.96
No vaccination^d^	Ref.		Ref.		Ref.		Ref.	Ref.	Ref.	Ref.
Vaccination^d^	NA		NA		NA		1.05	0.91–1.22	0.91	0.80–1.04

aOR: adjusted odds ratios using a mixed effect model adjusted for country as random intercept; AV: antiviral; ICU: intensive care unit; CI: confidence interval; NA: not analysed; Ref.: reference.

^a^ p < 0.05.

^b^ p < 0.001.

^c^ p < 0.01.

^d^ Only included in the age groups 60–79 and ≥ 80 years that are recommended for seasonal influenza vaccination.

Participating European Union countries: Austria, Belgium, Czechia, Ireland, Malta, the Netherlands, Portugal, Romania, Slovakia, Spain, Sweden.

The hospital ward-stratified analysis showed comparable results between ICU and non-ICU admitted patients, and the pooled analysis showed increasing age increased risk of death, and antiviral treatment initiation up to 7 days after symptom onset had a protective effect against death (Supplementary Table T4 showing the results of the multivariable logistic regression analysis stratified by ICU and non-ICU). Infection with influenza virus subtype A(H1N1)pdm09 was associated with increased risk of death in patients admitted to ICU.

### Underlying conditions and clinical complications

Data on underlying conditions were only available for 4,951 of 19,937 patients (24.8%). Of these, 1,153 patients (23.3%) had no underlying conditions or complications (Supplementary Tables T1, T5 and T7 showing descriptive characteristics of patients including underlying conditions and complications). The majority of cases with underlying conditions had one condition (67%, 2,554), with up to six different conditions reported in individual patients. Chronic lung disease was the most reported underlying condition (Supplementary Tables T1, T5 and T7). For patients with one or more underlying condition, 3,077 (81.0%) were discharged from hospital and 721 (19.0%) died. In contrast, only 8.9% (102/1,153) of patients with no underlying conditions died (p < 0.001). Data on clinical complications were reported for 17,150 patients and of these, 9.6% had no complications. Pneumonia was the most frequently reported clinical complication (75.7%), followed by ARDS (20.3%) and sepsis (5.1%).

The multivariable analysis including underlying conditions and complications confirmed the effect of increasing age, ICU admission and influenza virus type A infection on increased risk of death (Table 3). In addition, chronic liver disease (aOR 1.8, 95% CI: 1.1–2.9), HIV/immunosuppression (aOR 1.6, 95% CI: 1.1–2.1), ARDS (aOR 1.8, 95% CI: 1.3–2.5) and sepsis (aOR 3.3, 95% CI: 2.0–5.4) also increased the risk of dying. Antiviral treatment given within 48 hours compared with later was protective (aOR 0.8, 95% CI: 0.6–0.9), as was having chronic lung disease (aOR 0.7, 95% CI: 0.5–0.9). Pregnancy was not associated with mortality in women aged 14–49 years (Table 3).

**Table 3:** Risk of death by age, sex, underlying conditions, ICU admission status, time of treatment and vaccination status, 11 European Union countries, influenza seasons 2010/11–2019/20

Variable	All (n = 2,924)		Women 14-49 years (n = 1,520)	
	aOR	95% CI	aOR	95% CI
Male vs female (Ref.) sex	1.09	0.90–1.32	NA	NA
Age	1.03^a^	1.03–1.04	1.02	0.97–1.08
Influenza type A vs type B (Ref.) virus^b^	1.36^c^	1.01–1.83	2.72	0.77–9.55
AV treated within 48h vs later (Ref.)	0.72^d^	0.56–0.92	1.06	0.35–3.21
ICU vs non-ICU (Ref.) admission	4.59^a^	3.65–5.77	44.93^a^	7.81–258.43
Time between symptom onset and hospitalisation (days)	0.98	0.95–1.02	1.06	0.89–1.25
Vaccination vs no vaccination (Ref.)	0.87	0.67–1.12	NA	NA
Chronic lung disease vs no chronic lung disease (Ref.)	0.70^c^	0.53–0.92	NA	NA
Cancer vs no cancer (Ref.)	1.09	0.55–2.15	NA	NA
Diabetes vs no diabetes (Ref.)	0.83	0.64–1.08	0.14	0.01–1.41
Heart disease vs no heart disease (Ref.)	0.81	0.64–1.03	NA	NA
HIV/immunosuppression vs no HIV/immunosuppression (Ref.)	1.56^d^	1.14–2.13	3.26	0.95–11.22
Kidney disease vs no kidney disease (Ref.)	1.00	0.71–1.40	6.43 ^c^	1.22–33.85
Liver disease vs no liver disease (Ref.)	1.78^c^	1.11–2.87	1.93	0.23–16.23
Neuromuscular/cognitive vs no disorder (Ref.)	1.42	0.87–2.33	0.12	0.01–1.79
Obesity vs not obese (Ref.)	0.76	0.56–1.02	NA	NA
Pregnancy (n = 111) vs not pregnant (Ref.)	NA	NA	0.91	0.28–2.96
ARDS vs no ARDS (Ref.)	1.78^a^	1.27–2.49	2.86	0.53–15.38
Pneumonia vs no pneumonia (Ref.)	1.10	0.82–1.48	1.00	0.20–5.05
Sepsis vs no sepsis (Ref.)	3.32^a^	2.04–5.40	702.48^c^	3.20–150,000

aOR: adjusted odds ratio using a mixed effect model adjusted for country as random intercept.; ARDS: Acute respiratory distress syndrome; AV: antiviral; CI: confidence interval; HIV/immunos.: HIV infection or immunosuppression; ICU: Intensive care unit; NA: not analysed due to low numbers or being insignificant in univariable analysis, Ref.: reference.

^a^ p < 0.001.

^b^ only type A or B virus included due to low number of subtyped viruses.

^c^ p < 0.05.

^d^ p < 0.01.

Participating European Union countries: Austria, Belgium, Czechia, Ireland, Malta, the Netherlands, Portugal, Romania, Slovakia, Spain, Sweden.

### Sensitivity analysis

The matched analysis and recoded approach where all previously excluded cases with missing antiviral treatment information were recoded to ‘no treatment’ resulted in an increased (83,000 cases) dataset that overestimated the non-treated patients. Results are comparable to the primary model showing a lower likelihood of dying in patients treated with NAIs up to 7 days (within 48 hours for matched aOR 0.59, 95% CI: 0.46–0.77 and recoded aOR 0.75, 95% CI: 0.68- 0.82, respectively), and increased risk of death with A(H1N1)pdm09 virus infection (for matched aOR 1.57, 95% CI: 1.15–2.14 and recoded aOR 1.44, 95% CI: 1.34–1.54, respectively, Supplementary Tables T9, T11 and Figure F5 showing the flow chart and results of both logistic regression analyses).

Excluding the large Spanish data resulted in an analysis with only 1,694 observations, which also showed a lower risk of dying associated with early (within 48 hours) vs late antiviral treatment after symptom onset (aOR 0.68, 95% CI: 0.5-0.9, Supplementary Table T10).

The different analysis approaches also identified HIV/immunosuppression and chronic liver disease as associated risk factors for death (Supplementary Tables T10, T12 showing results of matched and recoded logistic regression analyses).

## Discussion

In this retrospective analysis of case-based surveillance data from almost 20,000 hospitalised patients with laboratory-confirmed influenza in 11 EU countries, we found that 28% of patients were admitted to ICU and 14% died. Antiviral treatment use was common but only 30% of patients received it within 48 hours after symptom onset and 83% within 48 hours after hospitalisation, which is in line with data from the United States (US) [22]. Antiviral use, when given within 48 hours and possibly up to 7 days after symptom onset, was shown to decrease the risk of dying after controlling for known confounders such as age, virus (sub)type, and time since illness onset. We showed that antiviral treatment had no major effect on fatal outcome of hospitalised younger people, who are less likely to die, but also less likely to receive antiviral treatment or be vaccinated. Vaccination did not show an effect on mortality, as discussed in a previous study [20]. However, vaccination may play a bigger role earlier in the course of the disease with other factors being more relevant when clinical conditions worsened.

Studies from the US have shown that antiviral (mostly oseltamivir) treatment in hospitalised children or adults reduced the length of stay when treated within 48 hours or 6–12 hours after symptom onset, respectively [15,22,23]. Another study identified that treatment up to 5 days after symptom onset improved survival in patients admitted to the ICU [24], which is in line with our findings. The protective effect of antiviral treatment in our analysis as indicated by the aOR is consistent with previous findings [13-15,25], also confirming that treatment later than 48 hours after symptom onset may have some protective effect against mortality [24]. Also, early hospital admission was shown to prevent severe outcome, possibly due to earlier and better treatment in hospital [26].

Age strongly influenced outcome, with older age having increased risk of dying after influenza infection. In addition, in the older age groups, NAI treatment lowered risk of dying more than in the younger age groups. Admission to ICU, liver disease, HIV/immunosuppression and clinical complications were confirmed factors associated with increased risk of death [20]. No detailed clinical information about the treatment of HIV infected persons, or the state of immunosuppression in patients was provided, and this should be a focus in follow-up studies to better understand treatment options. Clinicians seem to be aware of HIV/immunosuppression as a determinant for severe outcome indicated by the high proportion of these patients receiving NAI treatment similar to patients with cancer, while patients with liver disease, neuromuscular or neurocognitive conditions were less likely to receive NAIs.

Due to the COVID-19 pandemic and measures put in place to stop its spread, there has been a very low circulation of influenza in 2020, and as of March 2022, globally [27,28]. The lack of exposure and waning immunity to influenza in general, coupled with overall low vaccination rates, results in a population possibly more susceptible to influenza virus infection. Antiviral treatment remains an important treatment option to prevent severe disease and fatal outcome as shown in our analysis. This is particularly important for older age groups. However, barriers to timely treatment initiation or general use of antiviral treatment remain and are manifold, e.g. delayed or no laboratory confirmation of influenza, hesitation of clinicians to use NAIs due to concerns of effectiveness or lack of knowledge of clinical guidelines or relevant risk groups [29,30]. It is therefore important to confirm influenza early so that clinicians can initiate NAI treatment early to improve infectious disease control measures as well as outcomes of people at risk for severe disease.

### Limitations

Limitations of observational studies also apply to this analysis. First, the number of countries reporting changed over time and each country had a distinct health care system and likely variability in therapy. However, the core variables analysed (sex, age, ICU admission) were likely not collected or reported in substantially different ways as to bias the results. In addition, 83% of the data were from Spain, so the findings may not be representative of the situation in the EU as a whole and possibly have systematic bias in the recording of data. However, the analysis of chronic conditions is more balanced with Spain only contributing to 32% of the cases, and the sensitivity analysis resulted in comparable findings after adjusting for this. Second, only a quarter of the data had information on underlying disease, a known risk factor for influenza mortality, so multivariable models were probably underpowered. Third, a low number of patients (4.9%, 769/15,628) in the dataset received antiviral treatment after symptom onset but before hospital admission. Given that the median time to hospitalisation was 3.6 days, the dataset was not ideal for assessing the effect of very early antiviral use. Fourth, in some cases, when the date of symptom onset was not available, reporting physicians may have used another date, such as the date of laboratory testing. Laboratory testing and notification occur after symptom onset and this would underestimate the time between symptom onset to hospitalisation as well as to NAI treatment. For example, in Spain, onset symptoms reflect date of first clinical sign or symptom, and this information was available in most of the cases (> 95%). However, some cases may have been reported with date of testing instead if date of onset was not available. Fifth, underlying conditions or other variables that rely on clinical judgement may have been reported differently by individual clinicians in different countries. Sixth, most influenza viruses reported as type A unsubtyped may have been type A viruses testing negative for A(H1N1)pdm09 following the introduction of specific (H1N1)pdm09 PCR primers during the 2009 pandemic, but could also possibly be due to the increasing use of rapid antigen tests that only detect type A or B. However, another study previously showed that hospitalised patients with influenza A(H1N1)pdm09 had a higher risk of ICU admission or death than patients with influenza A(H3N2) or B, independent of other factors [31]. Seventh, different viral subtypes circulated during the study period, which could affect the pooled results (for example the B/Yamagata season 2017/18 that affected elderly people > 60 years and contributed to > 80% of the influenza type B cases). Eighth, death after hospital discharge was not possible to capture due to relying on hospital-based surveillance data. Despite these limitations that are common in surveillance data, all different multivariate models explored indicated that early antiviral use was independently protective against mortality.

### Conclusion

Surveillance data can be powerful for exploring effects in observational studies but also have limitations due to pooling of data from countries with different health care and monitoring structures. Our data underline the severity of influenza and possibly missed early treatment opportunities: 28% of hospitalised patients required intensive care, 14% died, but only 58% received antiviral treatment within the first 4 days and 30% within 48 hours as recommended. Our results support the role of antiviral treatment in hospitalised patients with laboratory-confirmed influenza to reduce the risk of in-hospital death when given early after influenza virus confirmation, but even possibly up to 7 days, when the 48-hour window after symptom onset has passed.

## Supplementary Data

727000 Supplementary Material

## References

[1] Nair H Brooks WA Katz M Roca A Berkley JA Madhi SA Global burden of respiratory infections due to seasonal influenza in young children: a systematic review and meta-analysis. Lancet. 2011;378(9807):1917-30. . Available from: http://www.ncbi.nlm.nih.gov/pubmed/22078723 10.1016/S0140-6736(11)61051-9 22078723

[2] Hayward AC Fragaszy EB Bermingham A Wang L Copas A Edmunds WJ Comparative community burden and severity of seasonal and pandemic influenza: results of the Flu Watch cohort study. Lancet Respir Med. 2014;2(6):445-54. 10.1016/S2213-2600(14)70034-7 24717637 PMC7164821

[3] World Health Organization (WHO). Global epidemiological surveillance standards for influenza. Geneva: WHO; 2013. [Accessed: 3 Jan 2023]. Available from: https://apps.who.int/iris/handle/10665/311261

[4] Abraham GM Morton JB Saravolatz LD . Baloxavir: A Novel Antiviral Agent in the Treatment of Influenza. Clin Infect Dis. 2020;71(7):1790-4. 10.1093/cid/ciaa107 32020174

[5] European Medicines Agency (EMA). Xofluza. Amsterdam: EMA. [Accessed: 3 Jan 2023]. Available from: https://www.ema.europa.eu/en/medicines/human/EPAR/xofluza

[6] European Medicines Agency (EMA). Relenza. Amsterdam: EMA. [Accessed: 3 Jan 2023]. Available from: https://www.ema.europa.eu/en/relenza

[7] European Medicines Agency (EMA). Tamiflu. Amsterdam: EMA. [Accessed: 3 Jan 2023]. Available from: https://www.ema.europa.eu/en/medicines/human/EPAR/tamiflu

[8] European Medicines Agency (EMA). Influenza vaccines – quality module. Amsterdam: EMA. [Accessed: 3 Jan 2023]. Available from: https://www.ema.europa.eu/en/influenza-vaccines-quality-module

[9] European medicines Agency (EMA). Update of EU recommendations for 2021-2022 seasonal flu vaccine composition. 2021. Amsterdam: EMA. [Accessed: 3 Jan 2023]. Available from: https://www.ema.europa.eu/en/news/update-eu-recommendations-2021-2022-seasonal-flu-vaccine-composition

[10] Hayden FG Asher J Cowling BJ Hurt AC Ikematsu H Kuhlbusch K Reducing influenza virus transmission: the value of antiviral treatment. Clin Infect Dis. 2022;74(3):532-40. 10.1093/cid/ciab625 34245250 PMC8834654

[11] Domínguez A Romero-Tamarit A Soldevila N Godoy P Jané M Martínez A Effectiveness of antiviral treatment in preventing death in severe hospitalised influenza cases over six seasons. Epidemiol Infect. 2018;146(7):799-808. 10.1017/S0950268818000663 29606178 PMC9184950

[12] Duwe SC Schmidt B Gärtner BC Timm J Adams O Fickenscher H Prophylaxis and treatment of influenza: options, antiviral susceptibility, and existing recommendations. GMS Infect Dis. 2021;9:Doc02. 34113534 10.3205/id000071 PMC8165743

[13] Muthuri SG Myles PR Venkatesan S Leonardi-Bee J Nguyen-Van-Tam JS . Impact of neuraminidase inhibitor treatment on outcomes of public health importance during the 2009-2010 influenza A(H1N1) pandemic: a systematic review and meta-analysis in hospitalized patients. J Infect Dis. 2013;207(4):553-63. 10.1093/infdis/jis726 23204175 PMC3549600

[14] Liu JW Lin SH Wang LC Chiu HY Lee JA . Comparison of antiviral agents for seasonal influenza outcomes in healthy adults and children: a systematic review and network meta-analysis. JAMA Netw Open. 2021;4(8):e2119151. 10.1001/jamanetworkopen.2021.19151 34387680 PMC8363918

[15] Hsu J Santesso N Mustafa R Brozek J Chen YL Hopkins JP Antivirals for treatment of influenza: a systematic review and meta-analysis of observational studies. Ann Intern Med. 2012;156(7):512-24. 10.7326/0003-4819-156-7-201204030-00411 22371849 PMC6679687

[16] Appiah GD Chaves SS Kirley PD Miller L Meek J Anderson E Increased antiviral treatment among hospitalized children and adults with laboratory-confirmed influenza, 2010-2015. Clin Infect Dis. 2017;64(3):364-7. 10.1093/cid/ciw745 28013261 PMC5480237

[17] Hooiveld M van de Groep T Verheij TJ van der Sande MA Verheij RA Tacken MA Prescription of antiviral drugs during the 2009 influenza pandemic: an observational study using electronic medical files of general practitioners in the Netherlands. BMC Pharmacol Toxicol. 2013;14(1):55. 10.1186/2050-6511-14-55 24143932 PMC3854647

[18] Doshi S Kamimoto L Finelli L Perez A Reingold A Gershman K Description of antiviral treatment among adults hospitalized with influenza before and during the 2009 pandemic: United States, 2005-2009. J Infect Dis. 2011;204(12):1848-56. 10.1093/infdis/jir648 22013219

[19] Snacken R Brown C . New developments of influenza surveillance in Europe. Euro Surveill. 2015;20(4):21020. 10.2807/ese.20.04.21020-en 25655056

[20] Adlhoch C Gomes Dias J Bonmarin I Hubert B Larrauri A Oliva Domínguez JA Determinants of fatal outcome in patients admitted to intensive care units with influenza, European Union 2009-2017. Open Forum Infect Dis. 2019;6(11):ofz462. . Available from: https://www.ncbi.nlm.nih.gov/pubmed/32258201 10.1093/ofid/ofz462 32258201 PMC7105050

[21] European Centre for Disease Prevention and Control (ECDC). Seasonal influenza vaccination and antiviral use in EU/EEA Member States. Stockholm: ECDC, 2018. Available from: https://www.ecdc.europa.eu/en/publications-data/seasonal-influenza-vaccination-antiviral-use-eu-eea-member-states

[22] Katzen J Kohn R Houk JL Ison MG . Early oseltamivir after hospital admission is associated with shortened hospitalization: a 5-year analysis of oseltamivir timing and clinical outcomes. Clin Infect Dis. 2019;69(1):52-8. 10.1093/cid/ciy860 30304487

[23] Campbell AP, Tokars JI, Reynolds S, Garg S, Kirley PD, Miller L, et al. Influenza antiviral treatment and length of stay. Pediatrics. 2021;148(4):e2021050417. 10.1542/peds.2021-050417 10.1542/peds.2021-050417 34470815

[24] Louie JK Yang S Acosta M Yen C Samuel MC Schechter R Treatment with neuraminidase inhibitors for critically ill patients with influenza A (H1N1)pdm09. Clin Infect Dis. 2012;55(9):1198-204. 10.1093/cid/cis636 22843781 PMC12362346

[25] Groeneveld GH Marbus SD Ismail N de Vries JJC Schneeberger P Oosterheert JJ Effectiveness of oseltamivir in reduction of complications and 30-day mortality in severe seasonal influenza infection. Int J Antimicrob Agents. 2020;56(5):106155. 10.1016/j.ijantimicag.2020.106155 32898685

[26] Myles P Nguyen-Van-Tam JS Semple MG Brett SJ Bannister B Read RC Differences between asthmatics and nonasthmatics hospitalised with influenza A infection. Eur Respir J. 2013;41(4):824-31. 10.1183/09031936.00015512 22903963 PMC3612580

[27] Olsen SJ Winn AK Budd AP Prill MM Steel J Midgley CM Changes in influenza and other respiratory virus activity during the COVID-19 pandemic - United States, 2020-2021. MMWR Morb Mortal Wkly Rep. 2021;70(29):1013-9. 10.15585/mmwr.mm7029a1 34292924 PMC8297694

[28] Adlhoch C Mook P Lamb F Ferland L Melidou A Amato-Gauci AJ Very little influenza in the WHO European Region during the 2020/21 season, weeks 40 2020 to 8 2021. Euro Surveill. 2021;26(11):2100221. 10.2807/1560-7917.ES.2021.26.11.2100221 33739256 PMC7976381

[29] Clark TW Beard KR Brendish NJ Malachira AK Mills S Chan C Clinical impact of a routine, molecular, point-of-care, test-and-treat strategy for influenza in adults admitted to hospital (FluPOC): a multicentre, open-label, randomised controlled trial. Lancet Respir Med. 2021;9(4):419-29. 10.1016/S2213-2600(20)30469-0 33285143 PMC9764870

[30] Brendish NJ Malachira AK Lillie PJ Clark TW . Neuraminidase inhibitor use in adults presenting to hospital with suspected influenza: A questionnaire-based survey of practice among hospital physicians. Clin Infect Pract. 2021;11:100075. 10.1016/j.clinpr.2021.100075

[31] Delgado-Sanz C Mazagatos-Ateca C Oliva J Gherasim A Larrauri A . Illness severity in hospitalized influenza patients by virus type and subtype, Spain, 2010-2017. Emerg Infect Dis. 2020;26(2):220-8. 10.3201/eid2602.181732 31961295 PMC6986827

