# Supplementary material for: Effect of neuraminidase inhibitor (oseltamivir) treatment on outcome of hospitalised influenza patients, surveillance data from 11 EU countries, 2010 to 2020
Source: Euro Surveill. 2023 Jan 26;28(4):2200340. doi: 10.2807/1560-7917.ES.2023.28.4.2200340 (PMC9881178; doi:10.2807/1560-7917.ES.2023.28.4.2200340)
Supplement: Supplementary Material [file 22-00340_ADLHOCH_Supplementary_material.pdf]

*****************************Supplement*************************************

Disclaimer:

This supplementary material is hosted by *Eurosurveillance* as supporting information alongside the article "Effect of neuraminidase inhibitor (oseltamivir) treatment on outcome of hospitalised influenza patients, surveillance data from 11 EU countries, 2010 to 2020", on behalf of the authors, who remain responsible for the accuracy and appropriateness of the content. The same standards for ethics, copyright, attributions and permissions as for the article apply. Supplements are not edited by *Eurosurveillance* and the journal is not responsible for the maintenance of any links or email addresses provided therein

Data inclusion and management of reporting underlying conditions through free text field:

A free text field on underlying other conditions was reviewed and information added into the predefined categories if considered relevant e.g., renal failure and chronic liver-kidney disease were added to chronic kidney disease entries. Also, free text entries (HIV, immunosuppression therapy, immunity violation, usage of immune suppressive treatment) were added to the variable HIV/immunosuppression. Several cancer forms were provided in the free text field that were also added to the overall variable cancer e.g., carcinoma bronchus or oesophagus, lymphoblastic leukaemia, breast cancer, medication for adenocarcinoma, etc.

Chronic underlying conditions obesity and morbid obesity were pooled and entries from the free text field added (class 2 obesity, obesity, tezaurismoza, obesity-sdr.pickwick). Neurocognitive and neuromuscular disease were pooled and other entries added into this new variable from the free text field (neurological disease, neurological disorder, chronic neurological disorder, multiple sclerosis, down syndrome, psychiatric disorder, trisomy 21, other myopathies, paraplegia decubitus, paratrophy, dorsal kyphosis, lumbar spondylosis, dystrophy, congenital malformation, weakness stature weighted). Asthma and chronic lung disease were pooled and free text entries were added (chronic obstructive pulmonary disease (COPD), chronic heart-lung disease, and chronic respiratory disease).

Table T1: Reporting countries and number of cases per season, total and by outcome

	Outcome					
	Recovered		Deceased		Total	
**Variable**	**N**	**(%)**	**N**	**(%)**	**N**	**(%)**
Influenza Seasons						
2010/11	648	(3.8%)	98	(3.5%)	746	(3.7%)
2011/12	154	(0.9%)	9	(0.3%)	163	(0.8%)
2012/13	205	(1.2%)	39	(1.4%)	244	(1.2%)
2013/14	1164	(6.8%)	172	(6.1%)	1336	(6.7%)
2014/15	828	(4.8%)	155	(5.5%)	983	(4.9%)
2015/16	2106	(12.3%)	293	(10.3%)	2399	(12.0%)
2016/17	1813	(10.6%)	332	(11.7%)	2145	(10.8%)
2017/18	4292	(25.1%)	739	(26.0%)	5031	(25.2%)
2018/19	3694	(21.6%)	713	(25.1%)	4407	(22.1%)
2019/20	2193	(12.8%)	290	(10.2%)	2483	(12.5%)
Reporting country						
Belgium	971	(5.7%)	48	(1.7%)	1019	(5.1%)
Czechia	302	(1.8%)	116	(4.1%)	418	(2.1%)
Spain	14241	(83.3%)	2252	(79.3%)	16493	(82.7%)
Ireland	7	(0.0%)	0	(0.0%)	7	(0.0%)
Malta	22	(0.1%)	1	(0.0%)	23	(0.1%)
The Netherlands	14	(0.1%)	1	(0.0%)	15	(0.1%)
Austria	29	(0.2%)	2	(0.1%)	31	(0.2%)
Portugal	54	(0.3%)	12	(0.4%)	66	(0.3%)
Romania	856	(5.0%)	199	(7.0%)	1055	(5.3%)
Slovakia	65	(0.4%)	39	(1.4%)	104	(0.5%)
Sweden	536	(3.1%)	170	(6.0%)	706	(3.5%)

Table T2: Reporting countries and number of cases per season, total and by antiviral treatment, 11 EU countries, 2010-2020

Variable	No treatment		Oseltamivir, Zanamivir or both or other		Total	
	n	(%)	n	(%)	N	(%)
Sex						
Female	2,125	(49.3%)	7,153	(45.8%)	9,278	(46.5%)
Male	2,188	(50.7%)	8,471	(54.2%)	10,659	(53.5%)
Age-group						
0-19	1,193	(27.7%)	1,184	(7.6%)	2,377	(11.9%)
20-39	221	(5.1%)	1,130	(7.2%)	1,351	(6.8%)
40-59	525	(12.2%)	3,464	(22.2%)	3,989	(20.0%)
60-79	1,183	(27.4%)	5,790	(37.1%)	6,973	(35.0%)
80+	1,191	(27.6%)	4,056	(26.0%)	5,247	(26.3%)
Influenza virus (B ref)						
Type B virus	1,171	(27.2%)	2,679	(17.1%)	3,850	(19.3%)
A(H1N1)pdm09	1,077	(25.0%)	4,264	(27.3%)	5,341	(26.8%)
A(H3N2)	1,187	(27.5%)	2,568	(16.4%)	3,755	(18.8%)
A unsubtyped	878	(20.4%)	6,113	(39.1%)	6,991	(35.1%)
Hospital unit						
Non-ICU	3,524	(81.7%)	10,868	(69.6%)	14,392	(72.2%)
ICU	789	(18.3%)	4,756	(30.4%)	5,545	(27.8%)
Fatal outcome						
Alive	3,687	(85.5%)	13,410	(85.8%)	17,097	(85.8%)
Died	626	(14.5%)	2,214	(14.2%)	2,840	(14.2%)
Treatment						
None	4,313	(100.0%)	0	(0.0%)	4,313	(21.6%)
Oseltamivir	0	(0.0%)	15,355	(98.3%)	15,355	(77.0%)
Zanamivir	0	(0.0%)	43	(0.3%)	43	(0.2%)
Both	0	(0.0%)	20	(0.1%)	20	(0.1%)
Other	0	(0.0%)	206	(1.3%)	206	(1.0%)
Timing of AV treatment						
No treatment	4,313	(100.0%)	0	(0.0%)	4,313	(21.9%)
Within 2days	0	(0.0%)	4,767	(30.9%)	4,767	(24.2%)
3-4days	0	(0.0%)	4,202	(27.3%)	4,202	(21.3%)
5-7days	0	(0.0%)	4,043	(26.2%)	4,043	(20.5%)
>7days	0	(0.0%)	2,406	(15.6%)	2,406	(12.2%)
Influenza Seasons						
10/11	102	(2.4%)	644	(4.1%)	746	(3.7%)
11/12	49	(1.1%)	114	(0.7%)	163	(0.8%)
12/13	72	(1.7%)	172	(1.1%)	244	(1.2%)
13/14	34	(0.8%)	1,302	(8.3%)	1,336	(6.7%)
14/15	73	(1.7%)	910	(5.8%)	983	(4.9%)
15/16	411	(9.5%)	1,988	(12.7%)	2,399	(12.0%)
16/17	472	(10.9%)	1,673	(10.7%)	2,145	(10.8%)
17/18	1,510	(35.0%)	3,521	(22.5%)	5,031	(25.2%)
18/19	1,105	(25.6%)	3,302	(21.1%)	4,407	(22.1%)
19/20	485	(11.2%)	1,998	(12.8%)	2,483	(12.5%)
Preconditions						
No precondition	687	(44.2%)	466	(13.7%)	1,153	(23.3%)
Any Precondition	869	(55.8%)	2,929	(86.3%)	3,798	(76.7%)
Number of preconditions						
0	687	(44.2%)	466	(13.7%)	1,153	(23.3%)
1	422	(27.1%)	2,132	(62.8%)	2,554	(51.6%)
2	273	(17.5%)	482	(14.2%)	755	(15.2%)
3	135	(8.7%)	217	(6.4%)	352	(7.1%)
4	28	(1.8%)	83	(2.4%)	111	(2.2%)
5	9	(0.6%)	12	(0.4%)	21	(0.4%)
6	2	(0.1%)	3	(0.1%)	5	(0.1%)
Number of complications						
0	1,078	(28.4%)	564	(4.2%)	1,642	(9.6%)
1	2,501	(66.0%)	11,127	(83.3%)	13,628	(79.5%)
2	183	(4.8%)	1,392	(10.4%)	1,575	(9.2%)
3	28	(0.7%)	268	(2.0%)	296	(1.7%)
4	0	(0.0%)	9	(0.1%)	9	(0.1%)
Country						
Belgium	927	(21.5%)	92	(0.6%)	1,019	(5.1%)
Czechia	23	(0.5%)	395	(2.5%)	418	(2.1%)
Spain	2,756	(63.9%)	13,737	(87.9%)	16,493	(82.7%)
Ireland	0	(0.0%)	7	(0.0%)	7	(0.0%)
Malta	17	(0.4%)	6	(0.0%)	23	(0.1%)
The Netherlands	7	(0.2%)	8	(0.1%)	15	(0.1%)
Austria	0	(0.0%)	31	(0.2%)	31	(0.2%)
Portugal	8	(0.2%)	58	(0.4%)	66	(0.3%)

Romania	433	(10.0%)	622	(4.0%)	1,055	(5.3%)
Slovakia	3	(0.1%)	101	(0.6%)	104	(0.5%)
Sweden	139	(3.2%)	567	(3.6%)	706	(3.5%)
	mean (SD)	median (IQR)	mean (SD)	median (IQR)	mean (SD)	median (IQR)
Age	52.2 (33.4538)	65 (70)	62.2 (67)	23.40564 (29)	60.0 (26.23602)	67 (32)
Days between symptom onset and hospitalisation	3.6 (3.067887)	3 (4)	3.7 (3.104651)	3 (3)	3.7 (3.097112)	3 (4)
Duration of hospitalisation	9.1 (12.11779)	6 (7)	13.5 (18.29081)	8 (11)	11.0 (15.24583)	7 (10)
Days between symptom onset and antiviral treatment	.		4.6 (3.488864)	4 (4)		

Figure F1: Time between onset of symptoms and hospitalisation for cases, by outcome and hospital ward, 2010-2020

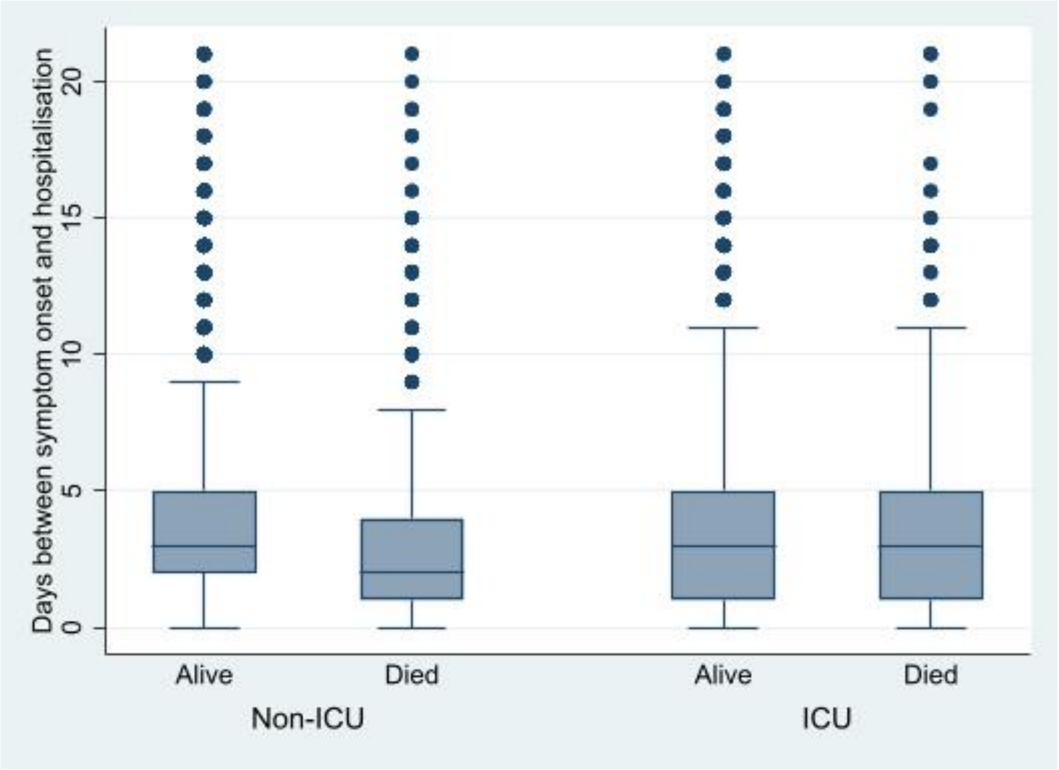

Figure F2: Time between onset of symptoms and onset of antiviral treatment by outcome and age-group in years, EU 2010-2020

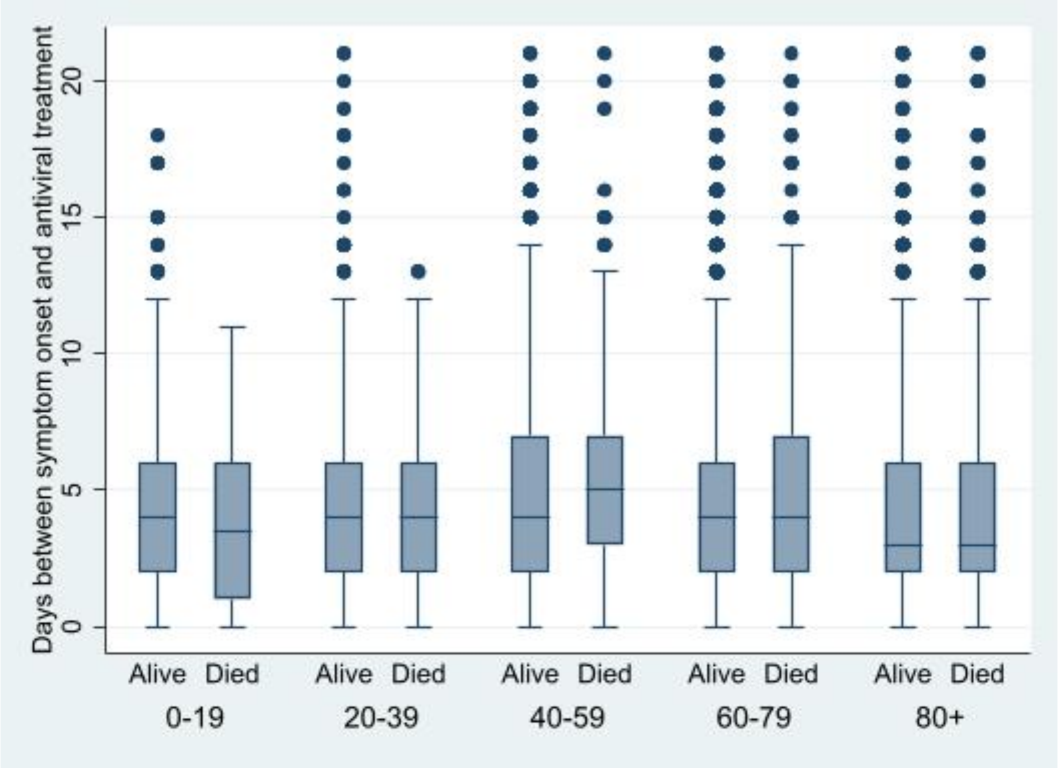

Figure F3: Time between onset of symptoms and onset of antiviral treatment by hospital ward, EU 2010-2020

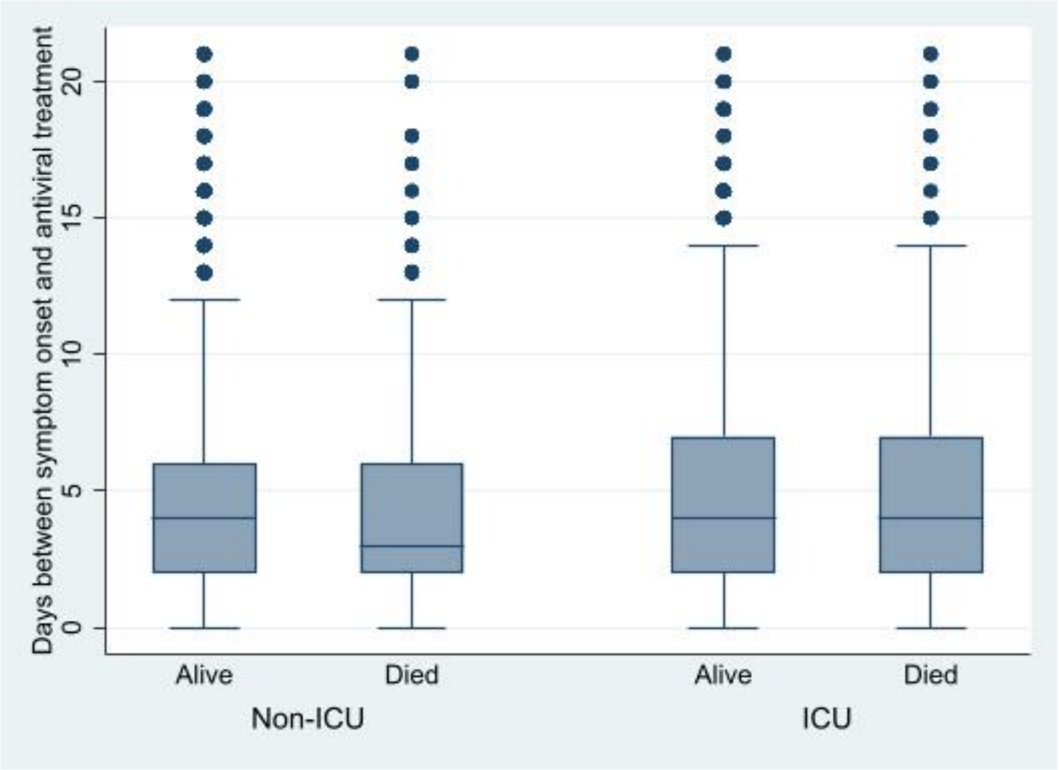

AV: antiviral; ICU: intensive care unit

Figure F4: Number and proportion of hospitalised influenza cases receiving antiviral treatment, by underlying precondition in 11 EU countries, 2010-2020

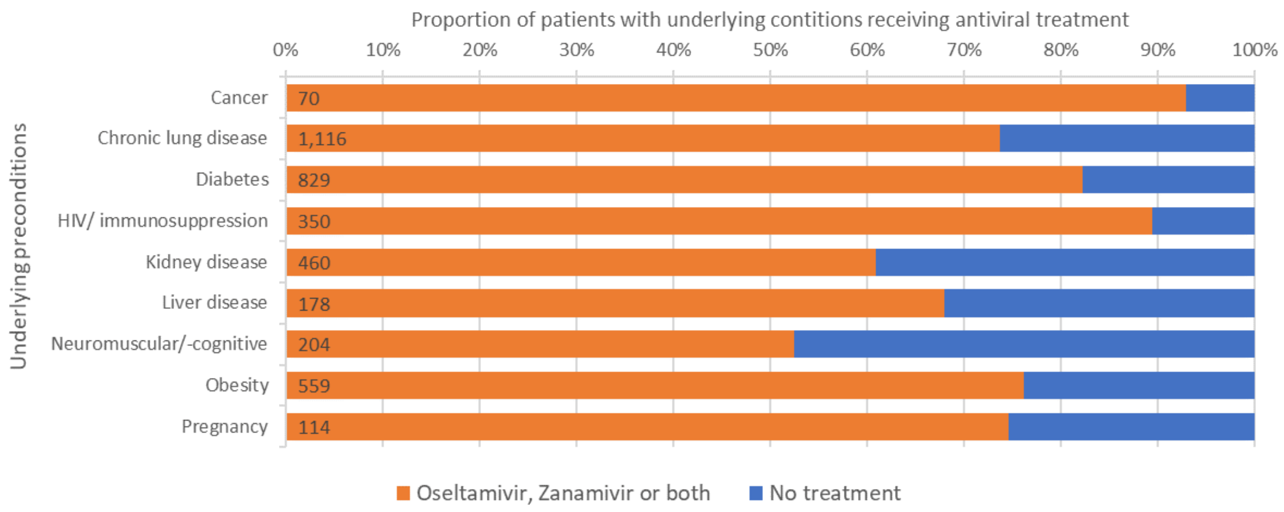

*Chronic lung disease covers asthma and other lung disease

Stratification by hospital unit

Table T3: Descriptive analysis stratified by hospital unit, 11 EU countries, 2010-2020

**Variable**	**Hospital unit**					
	**Non-ICU**		**ICU**		**Total**	
	**N**	**(%)**	**N**	**(%)**	**N**	**(%)**
Sex						
Female	6943	(48.2%)	2335	(42.1%)	9278	(46.5%)
Male	7449	(51.8%)	3210	(57.9%)	10659	(53.5%)
Age-group (years)						
0-19	1724	(12.0%)	653	(11.8%)	2377	(11.9%)
20-39	857	(6.0%)	494	(8.9%)	1351	(6.8%)
40-59	2406	(16.7%)	1583	(28.5%)	3989	(20.0%)
60-79	4637	(32.2%)	2336	(42.1%)	6973	(35.0%)
80+	4768	(33.1%)	479	(8.6%)	5247	(26.3%)
Influenza virus						
Type B	2930	(20.4%)	920	(16.6%)	3850	(19.3%)
A(H1N1)pdm09	3386	(23.5%)	1955	(35.3%)	5341	(26.8%)
A(H3N2)	2834	(19.7%)	921	(16.6%)	3755	(18.8%)
A unsubtyped	5242	(36.4%)	1749	(31.5%)	6991	(35.1%)
Influenza vaccination						
Not vaccinated	9246	(64.2%)	4274	(77.1%)	13520	(67.8%)
Vaccinated	5146	(35.8%)	1271	(22.9%)	6417	(32.2%)
Outcome						
Alive	12832	(89.2%)	4265	(76.9%)	17097	(85.8%)
Died	1560	(10.8%)	1280	(23.1%)	2840	(14.2%)
AV treatment						
No treatment	3524	(24.5%)	789	(14.2%)	4313	(21.6%)
Oseltamivir, Zanamivir or both	10868	(75.5%)	4756	(85.8%)	15624	(78.4%)
Treatment						
None	3524	(24.5%)	789	(14.2%)	4313	(21.6%)
Oseltamivir	10692	(74.3%)	4663	(84.1%)	15355	(77.0%)
Zanamivir	16	(0.1%)	27	(0.5%)	43	(0.2%)
Both	5	(0.0%)	15	(0.3%)	20	(0.1%)
Other	155	(1.1%)	51	(0.9%)	206	(1.0%)
Timing of AV treatment						
No treatment	3524	(24.8%)	789	(14.4%)	4313	(21.9%)
Within 2 days	3417	(24.0%)	1350	(24.6%)	4767	(24.2%)
3-4 days	3002	(21.1%)	1200	(21.8%)	4202	(21.3%)
5-7 days	2666	(18.7%)	1377	(25.1%)	4043	(20.5%)
>7 days	1628	(11.4%)	778	(14.2%)	2406	(12.2%)
Number of preconditions						
0	726	(24.6%)	427	(21.4%)	1153	(23.3%)
1	1600	(54.2%)	954	(47.8%)	2554	(51.6%)
2	367	(12.4%)	388	(19.4%)	755	(15.2%)
3	189	(6.4%)	163	(8.2%)	352	(7.1%)
4	59	(2.0%)	52	(2.6%)	111	(2.2%)
5	9	(0.3%)	12	(0.6%)	21	(0.4%)
6	4	(0.1%)	1	(0.1%)	5	(0.1%)
Number of complications						
0	1193	(9.5%)	449	(9.9%)	1642	(9.6%)
1	10525	(83.5%)	3103	(68.2%)	13628	(79.5%)
2	788	(6.3%)	787	(17.3%)	1575	(9.2%)
3	88	(0.7%)	208	(4.6%)	296	(1.7%)
4	6	(0.0%)	3	(0.1%)	9	(0.1%)
	mean (SD)	median (IQR)	mean (SD)	median (IQR)	mean (SD)	median (IQR)
Age	62.2 (27.0)	70 (33)	54.3 (23.1)	60 (27)	60.0 (26.2)	67 (32)
Time between symptom onset and AV treatment (days)	4.5 (3.5)	4 (4)	4.8 (3.5)	4 (5)	4.6 (3.5)	4 (4)
Time between onset of disease and hospitalisation (days)	3.7 (3.1)	3 (4)	3.7 (3.1)	3 (4)	3.7 (3.1)	3 (4)
Duration of hospitalisation (days)	9.0 (11.3)	6 (7)	15.4 (20.9)	10 (12)	11.0 (15.2)	7 (10)

AV: antiviral; ICU: Intensive care unit; EU countries: Austria, Belgium, Czechia, Ireland, Malta, The Netherlands, Portugal, Romania, Slovakia, Spain, Sweden

Table T4: Multivariable analysis stratified by hospital unit, 11 EU countries, 2010-2020

	Non-ICU		ICU	
**Variable**	**AOR**	**95% CI**	**AOR**	**95% CI**
Male sex	1.10	[0.98-1.23]	1.02	[0.89-1.16]
Age-group (years)				
0-19	0.30**	[0.13-0.71]	0.31***	[0.21-0.47]
20-39	Ref		Ref	
40-59	2.47**	[1.39-4.39]	1.35*	[1.02-1.78]
60-79	5.76***	[3.33-9.97]	2.50***	[1.91-3.27]
80+	18.42***	[10.67-31.80]	3.11***	[2.25-4.30]
Influenza virus				
Type B	Ref		Ref	
A(H1N1)pdm09	1.18	[0.99-1.41]	1.40**	[1.15-1.72]
A(H3N2)	1.02	[0.86-1.20]	0.96	[0.76-1.21]
A unsubtyped	0.81**	[0.69-0.94]	1.01	[0.82-1.24]
Timing of AV treatment				
No treatment	Ref		Ref	
Within 2 days	0.44***	[0.37-0.52]	0.67***	[0.53-0.85]
3-4 days	0.49***	[0.41-0.57]	0.77*	[0.61-0.97]
5-7 days	0.57***	[0.47-0.68]	0.75*	[0.59-0.94]
>7 days	0.81	[0.64-1.02]	1.28	[0.98-1.67]
Onset of symptoms to hospitalization (days)	1.10	[0.98-1.23]	1.02	[0.89-1.16]

AOR: adjusted odds ratio using a mixed effect model adjusted for country as random intercept; 95% CI: 95% confidence interval. * $p < 0.05$, ** $p < 0.01$, *** $p < 0.001$; EU countries: Austria, Belgium, Czechia, Ireland, Malta, The Netherlands, Portugal, Romania, Slovakia, Spain, Sweden; ICU: Intensive care unit;

Analysis including underlying conditions and clinical complications

Table T5: Descriptive table of number of underlying conditions and complications, 11 EU countries, 2010-2020

Variable	Outcome					
	Recovered		Deceased		Total	
	N	(%)	N	(%)	N	(%)
Conditions						
None	1051	(25.5%)	102	(12.4%)	1153	(23.3%)
Any	3077	(74.5%)	721	(87.6%)	3798	(76.7%)
Number of conditions						
0	1051	(25.5%)	102	(12.4%)	1153	(23.3%)
1	2128	(51.6%)	426	(51.8%)	2554	(51.6%)
2	581	(14.1%)	174	(21.1%)	755	(15.2%)
3	263	(6.4%)	89	(10.8%)	352	(7.1%)
4	84	(2.0%)	27	(3.3%)	111	(2.2%)
5	17	(0.4%)	4	(0.5%)	21	(0.4%)
6	4	(0.1%)	1	(0.1%)	5	(0.1%)
Number of complications						
0	1492	(10.0%)	150	(6.6%)	1642	(9.6%)
1	12052	(81.0%)	1576	(69.2%)	13628	(79.5%)
2	1196	(8.0%)	379	(16.6%)	1575	(9.2%)
3	132	(0.9%)	164	(7.2%)	296	(1.7%)
4	1	(0.0%)	8	(0.4%)	9	(0.1%)

EU countries: Austria, Belgium, Czechia, Ireland, Malta, The Netherlands, Portugal, Romania, Slovakia, Spain, Sweden

Table T6: Descriptive analysis for underlying conditions and complications, by country, 11 EU countries, 2010-2020

	Number of underlying conditions								
Country	0	1	2	3	4	5	6	Total	%
Austria	16	15	0	0	0	0	0	31	0.6
Belgium	391	306	187	100	27	7	1	1,019	20.6
Czechia	21	105	136	83	39	7	1	392	7.9
Ireland	0	5	1	0	0	0	0	6	0.1
Malta	16	7	0	0	0	0	0	23	0.5
Netherlands	1	10	3	1	0	0	0	15	0.3
Portugal	20	29	17	0	0	0	0	66	1.3
Romania	382	306	217	102	39	6	3	1,055	21.3
Slovakia	2	70	0	0	0	0	0	72	1.5
Spain	0	1,566	0	0	0	0	0	1,566	31.6
Sweden	304	135	194	66	6	1	0	706	14.3
Total	1,153	2,554	755	352	111	21	5	4,951	

ARDS: Acute respiratory distress syndrome; AV: antiviral; ICU: Intensive care unit; HIV/immunos.: HIV infection or immunosuppression

Table T7: Descriptive analysis for underlying conditions and complications, by outcome, 11 EU countries, 2010-2020

	**Alive**		**Died**		**Total**	
	**N**	**(%)**	**N**	**(%)**	**N**	**(%)**
Asthma						
No	3963	(96.0)	800	(97.2)	4763	(96.2)
Yes	165	(4.0)	23	(2.8)	188	(3.8)
Cancer						
No	4078	(98.8)	803	(97.6)	4881	(98.6)
Yes	50	(1.2)	20	(2.4)	70	(1.4)
Diabetes						
No	3466	(84.0)	656	(79.7)	4122	(83.3)
Yes	662	(16.0)	167	(20.3)	829	(16.7)
HIV/immunosuppression						
No	3876	(93.9)	725	(88.1)	4601	(92.9)
Yes	252	(6.1)	98	(11.9)	350	(7.1)
Kidney disease						
No	3755	(91.0)	736	(89.4)	4491	(90.7)
Yes	373	(9.0)	87	(10.6)	460	(9.3)
Liver disease						
No	3996	(96.8)	777	(94.4)	4773	(96.4)
Yes	132	(3.2)	46	(5.6)	178	(3.6)
Lung disease						
No	3300	(79.9)	682	(82.9)	3982	(80.4)
Yes	828	(20.1)	141	(17.1)	969	(19.6)
Neurocognitive						
No	4110	(99.6)	815	(99.0)	4925	(99.5)
Yes	18	(0.4)	8	(1.0)	26	(0.5)
Neuromuscular						
No	3982	(96.5)	787	(95.6)	4769	(96.3)
Yes	146	(3.5)	36	(4.4)	182	(3.7)
Obesity						
No	3686	(89.3)	706	(85.8)	4392	(88.7)
Yes	442	(10.7)	117	(14.2)	559	(11.3)
Pregnancy						
No	,022	(97.4)	815	(99.0)	4837	(97.7)
Yes	106	(2.6)	8	(1.0)	114	(2.3)
Chronic lung disease						

No	3168	(76.7)	667	(81.0)	3835	(77.5)
Yes	960	(23.3)	156	(19.0)	1,116	(22.5)
Neuromuscular/-cognitive						
No	3966	(96.1)	781	(94.9)	4747	(95.9)
Yes	162	(3.9)	42	(5.1)	204	(4.1)
ARDS						
No	12112	(81.4)	1561	(68.6)	13673	(79.7)
Yes	2761	(18.6)	716	(31.4)	3477	(20.3)
Bronchitis						
No	14842	(99.8)	2264	(99.4)	17106	(99.7)
Yes	31	(0.2)	13	(0.6)	44	(0.3)
Encephalitis						
No	14840	(99.8)	2270	(99.7)	17110	(99.8)
Yes	33	(0.2)	7	(0.3)	40	(0.2)
Myocarditis						
No	14836	(99.8)	2260	(99.3)	17096	(99.7)
Yes	37	(0.2)	17	(0.7)	54	(0.3)
Pneumonia						
No	3404	(22.9)	763	(33.5)	4167	(24.3)
Yes	11469	(77.1)	1514	(66.5)	12983	(75.7)
Sepsis						
No	14533	(97.7)	1749	(76.8)	16282	(94.9)
Yes	340	(2.3)	528	(23.2)	868	(5.1)

ARDS: Acute respiratory distress syndrome; EU countries: Austria, Belgium, Czechia, Ireland, Malta, The Netherlands, Portugal, Romania, Slovakia, Spain, Sweden

Table T8: Reported underlying conditions and complications, by antiviral treatment, 11 EU countries, 2010-2020

Variable	No treatment		Treatment<br>Oseltamivir, Zanamivir or both or other		Total	
	n	(%)	n	(%)	N	(%)
Asthma						
No	1,459	(93.8)	3,304	(97.3)	4,763	(96.2)
Yes	97	(6.2)	91	(2.7)	188	(3.8)
Cancer						
No	1,551	(99.7)	3,330	(98.1)	4,881	(98.6)
Yes	5	(0.3)	65	(1.9)	70	(1.4)
Diabetes						
No	1,409	(90.6)	2,713	(79.9)	4,122	(83.3)
Yes	147	(9.4)	682	(20.1)	829	(16.7)
HIV/ immunosuppression						
No	1,519	(97.6)	3,082	(90.8)	4,601	(92.9)
Yes	37	(2.4)	313	(9.2)	350	(7.1)
Kidney disease						
No	1,376	(88.4)	3,115	(91.8)	4,491	(90.7)
Yes	180	(11.6)	280	(8.2)	460	(9.3)
Liver disease						
No	1,499	(96.3)	3,274	(96.4)	4,773	(96.4)
Yes	57	(3.7)	121	(3.6)	178	(3.6)
Lung disease						
No	1,331	(85.5)	2,651	(78.1)	3,982	(80.4)
Yes	225	(14.5)	744	(21.9)	969	(19.6)
Neurocognitive						
No	1,548	(99.5)	3,377	(99.5)	4,925	(99.5)
Yes	8	(0.5)	18	(0.5)	26	(0.5)
Neuromuscular						
No	1,466	(94.2)	3,303	(97.3)	4,769	(96.3)
Yes	90	(5.8)	92	(2.7)	182	(3.7)
Obesity						
No	1,423	(91.5)	2,969	(87.5)	4,392	(88.7)
Yes	133	(8.5)	426	(12.5)	559	(11.3)
Pregnancy						
No	1,527	(98.1)	3,310	(97.5)	4,837	(97.7)
Yes	29	(1.9)	85	(2.5)	114	(2.3)
Chronic lung disease						
No	1,263	(81.2)	2,572	(75.8)	3,835	(77.5)
Yes	293	(18.8)	823	(24.2)	1,116	(22.5)
Neuromusculus/-cognative						
No	1,459	(93.8)	3,288	(96.8)	4,747	(95.9)
Yes	97	(6.2)	107	(3.2)	204	(4.1)
ARDS						
No	3249	(85.7)	10424	(78.0)	13673	(79.7)
Yes	541	(14.3)	2936	(22.0)	3477	(20.3)
Bronchitis						
No	3785	(99.9)	13321	(99.7)	17106	(99.7)
Yes	5	(0.1)	39	(0.3)	44	(0.3)
Encephalitis						
No	3784	(99.8)	13326	(99.7)	17110	(99.8)
Yes	6	(0.2)	34	(0.3)	40	(0.2)
Myocarditis						
No	3785	(99.9)	13311	(99.6)	17096	(99.7)
Yes	5	(0.1)	49	(0.4)	54	(0.3)
Pneumonia						
No	1592	(42.0)	2575	(19.3)	4167	(24.3)
Yes	2198	(58.0)	10785	(80.7)	12983	(75.7)
Sepsis						
No	3637	(96.0)	12645	(94.6)	16282	(94.9)
Yes	153	(4.0)	715	(5.4)	868	(5.1)

ARDS: Acute respiratory distress syndrome; AV: antiviral; ICU: Intensive care unit; HIV/immunos.: HIV infection or immunosuppression; EU countries: Austria, Belgium, Czechia, Ireland, Malta, The Netherlands, Portugal, Romania, Slovakia, Spain, Sweden

Sensitivity analysis 1

We created a 1:1 matched dataset for antiviral treatment and used age, sex, ICU admission and reporting country as matching variables. The matched dataset was analysed by a conditional logistic regression for the outcome mortality.

Table T9: Results of the matched (age, sex, ICU admission and reporting country) conditional logistic regression for fatal outcome (N= 1,638 observations), 11 EU countries, 2010-2020

Variable	AOR	95% CI
Timing of AV treatment		
No treatment	Ref	
Within 2days	0.59***	[0.46-0.77]
3-4days	0.66**	[0.50-0.87]
5-7days	0.59***	[0.43-0.80]
>7days	1.15	[0.76-1.73]
Influenza virus		
B	Ref	
A(H1N1)pdm09	1.57**	[1.15-2.14]
A(H3N2)	1.19	[0.88-1.61]
A unsubtyped	1.12	[0.83-1.51]
Days between symptom onset and hospitalisation	0.91***	[0.88-0.95]

AOR: adjusted odds ratio using a mixed effect model adjusted for country as random intercept; 95% CI: 95% confidence interval; AV: antiviral;

Sensitivity analysis 2

We excluded all Spanish data and applied the same mixed effect logistic regression model adjusted for country as random intercept of a dataset with totally 3,444 cases.

Table T10: Results of the matched (age, sex, ICU admission and reporting country) conditional logistic regression for fatal outcome (N= 1,694 observations), 10 EU countries (excluding Spain), 2010-2020

Variable	AOR	95% CI
Male sex	1.16	[0.91-1.49]
Age	1.03***	[1.02-1.04]
Influenza type (B ref)	1.47*	[1.02-2.10]
AV treated 48 vs later	0.68*	[0.50-0.93]
ICU admission	5.78***	[4.15-8.05]
Onset of symptoms to hospitalisation (days)	0.98	[0.93-1.03]
Vaccination	0.79	[0.53-1.16]
Chronic lung disease	0.76	[0.51-1.16]
Cancer	1.08	[0.54-2.18]
Diabetes	0.93	[0.65-1.32]
Heart disease	0.85	[0.64-1.13]
HIV/ immunosuppression	1.73**	[1.17-2.56]
Kidney disease	0.74	[0.48-1.15]
Liver disease	1.68	[0.87-3.22]
Neuromuscular/-cognitive	1.39	[0.84-2.31]
Obesity	0.70*	[0.50-0.98]
ARDS	2.70***	[1.75-4.16]
Pneumonia	0.87	[0.61-1.24]
Sepsis	2.86***	[1.56-5.24]

AOR: adjusted odds ratio using a mixed effect model adjusted for country as random intercept; 95% CI: 95% confidence interval. * $p < 0.05$, ** $p < 0.01$, *** $p < 0.001$; ARDS: Acute respiratory distress syndrome; AV: antiviral; ICU: Intensive care unit; HIV/immunos.: HIV infection or immunosuppression; NA: not analyzed due to low numbers or being insignificant in univariable analysis. EU countries: Austria, Belgium, Czechia, Ireland, Malta, The Netherlands, Portugal, Romania, Slovakia, Sweden

Sensitivity analysis 3

We used the 83,880 cases with known age, sex, viral (sub)type, hospital unit type and outcome to recode cases reported with missing information on antiviral treatment previously excluded from the final analysis.

Figure F5: Flow chart of the included and excluded cases for the sensitivity analysis, EU 2010-2020

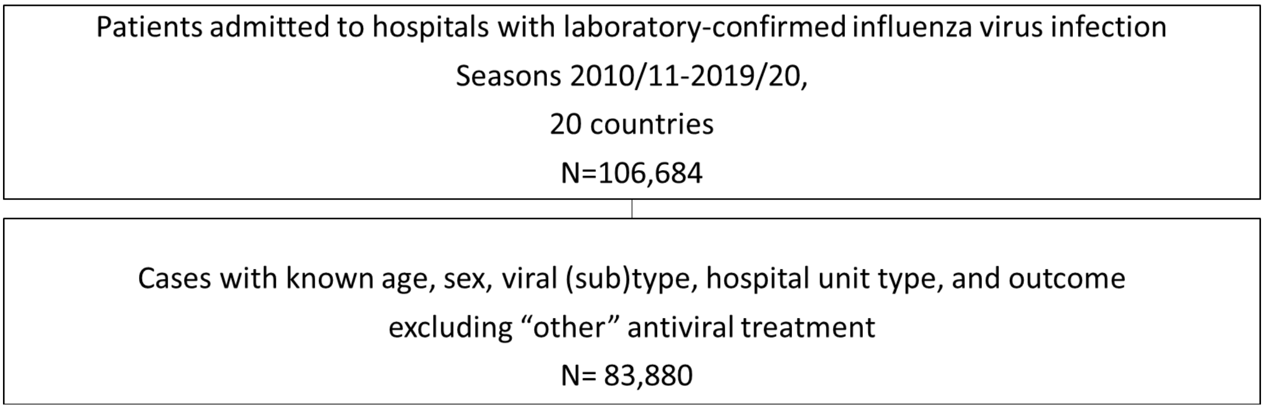

This created a dataset with 22,477 cases with antiviral treatment (no time exclusion) and 61,403 cases with "no treatment". EU countries: Austria, Belgium, Czechia, Denmark, Estonia, Finland, France, Ireland, Malta, The Netherlands, Portugal, Romania, Slovakia, Spain, Sweden.

Table T11: Results of the multivariable analysis full dataset with recoded 'missing data' for antiviral treatment as 'no treatment' for fatal outcome, 15 EU countries, 2010-2020 (N=80,773)

Variable	AOR	95% CI
Male sex	1.08**	[1.03- 1.13]
Age-group		
0-19	0.41***	[0.35- 0.50]
40-59	1.74***	[1.53- 1.98]
60-79	3.19***	[2.82- 3.60]
80+	7.14***	[6.30- 8.10]
Influenza virus		
A(H1N1)pdm09	1.44***	[1.34- 1.54]
A(H3N2)	1.04	[0.97- 1.12]
A unsubtyped	0.90***	[0.84- 0.96]
AV timing of treatment		
within 2days	0.75***	[0.68- 0.82]
3-4days	0.66***	[0.59- 0.73]
5-7days	0.57***	[0.52- 0.64]
>7days	0.73***	[0.65- 0.83]
ICU admission	4.12***	[3.86- 4.39]

AOR: adjusted odds ratio using a mixed effect model adjusted for country as random intercept; 95% CI: 95% confidence interval. * $p < 0.05$, ** $p < 0.01$, *** $p < 0.001$;

Table T12: Results of the multivariable mixed effect logistic regression of the full dataset with recoded missing cases for antiviral treatment as "no treatment" and known underlying conditions for fatal outcome, 15 EU countries, 2010-2020

(N=7,005)	AOR	95% CI
Male sex	1.18*	[1.03- 1.35]
Age-group		
0-19	0.55**	[0.37- 0.81]
40-59	1.54**	[1.16- 2.05]
60-79	2.51***	[1.89- 3.33]
80+	4.88***	[3.54- 6.74]
Influenza virus		
A(H1N1)pdm09	1.55***	[1.26- 1.91]
A(H3N2)	0.99	[0.79- 1.25]
A unsubtyped	0.99	[0.80- 1.24]
AV timing of treatment		
within 2days	0.82	[0.65- 1.03]
3-4days	1.07	[0.84- 1.36]
5-7days	0.96	[0.75- 1.22]
>7days	1.42*	[1.09- 1.86]
ICU admission	3.93***	[3.29- 4.69]
Vaccination	1.05	[0.89- 1.24]
Chronic lung disease	0.86	[0.70- 1.05]
Cancer	1.35	[0.82- 2.22]
Diabetes	0.92	[0.76- 1.12]
Heart disease	0.96	[0.80- 1.16]
HIV/immunosuppression	1.68***	[1.29- 2.18]
Kidney disease	1.18	[0.92- 1.52]
Liver disease	1.72**	[1.21- 2.46]
Neuromuscular/-cognitive	1.41	[0.98- 2.02]
Obesity	0.91	[0.74- 1.10]
ARDS	1.62***	[1.27- 2.06]
Pneumonia	1.00	[0.81- 1.24]
Sepsis	3.91***	[2.79- 5.48]

AOR: adjusted odds ratio using a mixed effect model adjusted for country as random intercept; 95% CI: 95% confidence interval. * $p < 0.05$, ** $p < 0.01$, *** $p < 0.001$; ARDS: Acute respiratory distress syndrome; AV: antiviral; ICU: Intensive care unit; HIV/immunos.: HIV infection or immunosuppression; NA: not analyzed due to low numbers or being insignificant in univariable analysis.

Study protocol

The study protocol was developed for an ad hoc analysis of the reported surveillance data together with the influenza network in the EU/EEA countries to be presented at the Options X conference. The analysis was then considered valuable for a peer-review publication and further post hoc developed with the feedback of the co-authors while writing. Post hoc analyses were related to the age-stratification, inclusion of the vaccination into the models only for the elderly where a vaccination recommendation exists and the use of different PS approaches to investigate and address possible biases in treatment.

PDF

Surveillance studyProtocol_AVtrea
